# Lamin A Rod Domain Mutants Target Heterochromatin Protein 1α and β for Proteasomal Degradation by Activation of F-Box Protein, FBXW10

**DOI:** 10.1371/journal.pone.0010620

**Published:** 2010-05-13

**Authors:** Pankaj Chaturvedi, Veena K. Parnaik

**Affiliations:** Centre for Cellular and Molecular Biology (CSIR), Hyderabad, India; Tulane University Health Sciences Center, United States of America

## Abstract

**Background:**

Lamins are major structural proteins of the nucleus and contribute to the organization of various nuclear functions. Mutations in the human lamin A gene cause a number of highly degenerative diseases, collectively termed as laminopathies. Cells expressing lamin mutations exhibit abnormal nuclear morphology and altered heterochromatin organization; however, the mechanisms responsible for these defects are not well understood.

**Methodology and Principal Findings:**

The lamin A rod domain mutants G232E, Q294P and R386K are either diffusely distributed or form large aggregates in the nucleoplasm, resulting in aberrant nuclear morphology in various cell types. We examined the effects of these lamin mutants on the distribution of heterochromatin protein 1 (HP1) isoforms. HeLa cells expressing these mutants showed a heterogeneous pattern of HP1α and β depletion but without altering HP1γ levels. Changes in HP1α and β were not observed in cells expressing wild-type lamin A or mutant R482L, which assembled normally at the nuclear rim. Treatment with proteasomal inhibitors led to restoration of levels of HP1 isoforms and also resulted in stable association of lamin mutants with the nuclear periphery, rim localization of the inner nuclear membrane lamin-binding protein emerin and partial improvement of nuclear morphology. A comparison of the stability of HP1 isoforms indicated that HP1α and β displayed increased turnover and higher basal levels of ubiquitination than HP1γ. Transcript analysis of components of the ubiquitination pathway showed that a specific F-box protein, FBXW10 was induced several-fold in cells expressing lamin mutants. Importantly, ectopic expression of FBXW10 in HeLa cells led to depletion of HP1α and β without alteration of HP1γ levels.

**Conclusions:**

Mislocalized lamins can induce ubiquitin-mediated proteasomal degradation of certain HP1 isoforms by activation of FBXW10, a member of the F-box family of proteins that is involved in E3 ubiquitin ligase activity.

## Introduction

Lamins are type V intermediate filament proteins that are the major structural proteins of the nucleus in metazoan cells. Lamins form a filamentous meshwork underlying the inner nuclear membrane that extends into the nucleoplasm. Two types of lamins are found in most species. The B-type lamins B1 and B2 are expressed in all somatic cells and are coded by separate genes. The A-type lamins A and C are encoded by a single lamin A gene through alternative splicing and their expression is detectable in several differentiated cell types. Lamins are important for maintenance of nuclear shape and integrity and are involved in the organization of nuclear functions such as DNA replication and transcription; the A-type lamins have also been proposed to play important roles in cell differentiation and gene regulatory pathways. Mutations in the human lamin A gene (*LMNA*) are associated with at least 15 highly degenerative, inherited diseases collectively termed laminopathies. The majority of mutations in *LMNA* are missense mutations, though small deletions and truncations have also been identified. Most of the mapped mutations cause Emery-Dreifuss muscular dystrophy (EMD) and dilated cardiomyopathy while other mutations are associated with progerias and lipodystrophies such as familial partial lipodystrophy (FPLD) [1]–[6].

A number of studies suggest that lamins are important for chromatin organization, with findings including in vitro binding to DNA, immunolocalization of the lamina at the nuclear periphery in close contact with chromatin, and association of lamins with chromatin-binding proteins such as barrier-to-autointegration factor (BAF) and lamina-associated polypeptides (LAPs) like LAP2α [5], [7], [8]. This is further supported by evidence of abnormalities in heterochromatin organization in cells from laminopathic patients, which are well documented in cells from patients with Hutchinson-Gilford progeria syndrome (HGPS), an inherited disease arising from a splicing defect in pre-lamin A [9]–[14], and those with mandibuloacral dysplasia type A (MAD-A) due to a R527H mutation in *LMNA* [15]. Mature lamin A is normally derived from pre-lamin A by extensive post-translational modifications, namely farnesylation of cysteine in the C-terminal–CSIM motif, followed by proteolytic cleavage of the last three amino acids, carboxymethylation of the farnesylated cysteine and proteolytic cleavage of the C-terminal 15 amino acids. On the other hand, HGPS cells express aberrantly processed lamin A, termed LAΔ50 or progerin, in which the farnesylated C-terminus is retained. Abnormal nuclear morphology, defective nuclear envelopes and disorganization of heterochromatin are also evident in laminopathic cells expressing EMD mutations that do not lead to retention of the farnesylated C-terminus [16], [17]. Interestingly, alterations in nuclear positioning of specific chromosomes have been observed in laminopathic cells [18]. Ectopic expression of disease-causing lamin mutants in cultured cells also causes abnormal nuclear morphology, altered lamin assembly and nuclear envelope defects [16], [19]–[22]. Like other intermediate filament proteins, lamin A is comprised of a rigid rod domain flanked by globular N-terminal and C-terminal domains [23]. As the rod domain of the protein is involved in coiled-coil interactions between lamin dimers, mutations in this segment are more likely to affect lamin assembly.

In the present study, we analyzed the levels and stability of the heterochromatin marker, heterochromatin protein 1 (HP1) in HeLa cells expressing disease-causing lamin A mutants. As our data showed a substantial loss of specific isoforms of HP1 in cells expressing the rod domain mutants G232E, Q294P and R386K that form intranuclear aggregates, we determined whether this downregulation was due to proteasomal degradation by using proteasomal inhibitors, analyzed the distribution of lamin A in treated cells and studied the turnover rates and ubiquitination levels of HP1 isoforms. Furthermore, we compared the expression levels of a number of components of the ubiquitination pathway and studied the effects of overexpression of one F-box protein, FBXW10 that was highly induced in mutant-expressing cells. Our findings indicate that lamin mutants can induce ubiquitin-dependent proteolysis of specific HP1 isoforms by activation of a distinct F-box protein that is involved in E3 ubiquitin ligase activity.

## Results

### Expression of Lamin A Mutants Causes Depletion of Hp1α and β

**Figure 1:**
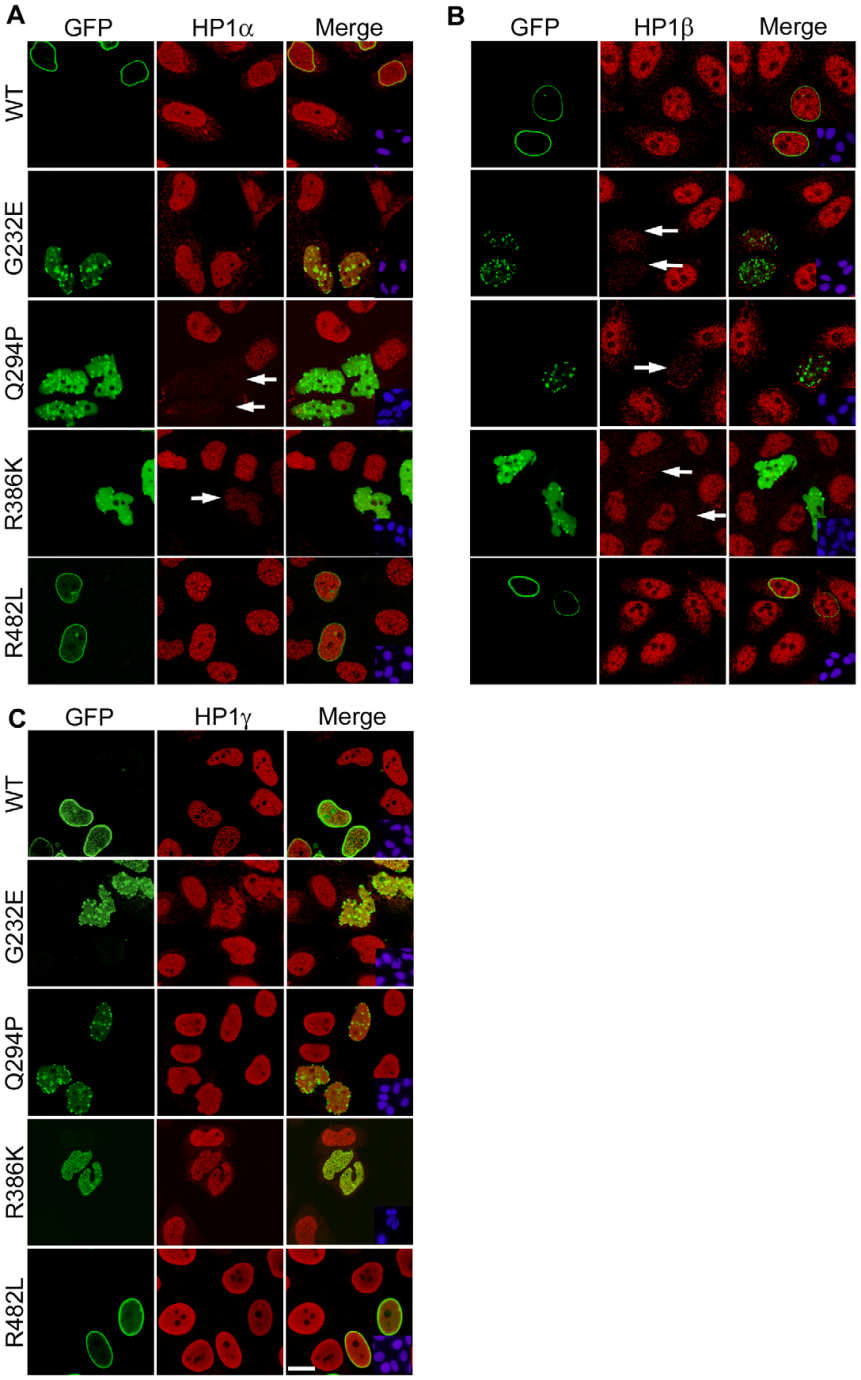
Localization of HP1α, β and γ in HeLa cells expressing lamin mutants. (**A–C**) HeLa cells transiently transfected with the indicated GFP-tagged lamin constructs were stained with antibodies to HP1α, β or γ respectively and counterstained with DAP1 (insets). Arrows indicate transfected cells depleted of HP1α or β. Bar, 10 µm.

We examined the expression of heterochromatin markers in HeLa cells expressing GFP fused constructs of wild-type lamin A, the rod domain EMD mutants G232E, Q294P and R386K or the tail domain FPLD mutant R482L. Our previous studies with these constructs showed that wild-type GFP-lamin A and R482L were localized at the nuclear periphery in a typical rim pattern, whereas G232E, Q294P and R386K did not assemble at the nuclear periphery but mislocalized to the nucleoplasm where they formed varying numbers of aggregates, resulting in disruption of the lamina and abnormal nuclear morphology [22], [24]. Mammalian HP1 exists in three isoforms, α, β and γ. HP1α and β are enriched in heterochromatic foci, whereas HP1γ has been detected in both euchromatin and heterochromatin [25], [26]. HeLa cells expressing wild-type lamin A or R482L displayed normal patterns of expression of HP1α, β and γ in >90% of cells (Figure 1). However, HP1α was reduced in cells expressing the highly deleterious mutation R386K and normal levels were observed only in approximately 40% of cells, whereas 60% of cells expressing Q294P and 70% of cells expressing G232E showed normal levels of HP1α (Figure 1A and Table 1, untreated). Importantly, the majority of cells expressing G232E, Q294P or R386K showed nearly complete depletion of HP1β (Figure 1B and Table 1, untreated). On the other hand, the distribution of HP1γ was not significantly affected in cells expressing lamin mutants (Figure 1C). Similar results were obtained with NIH3T3 mouse fibroblasts (data not shown). In human cells HP1α and HP1β proteins are diffusely distributed and visibly enriched in only a few heterochromatic foci.

**Table 1:** Effect of proteasomal inhibitors on HP1α and HP1β protein levels.

Construct	Untreated	Untreated	MG132-treated	MG132-treated	Lactacystin-treated	Lactacystin-treated
	HP1α	HP1β	HP1α	HP1β	HP1α	HP1β
WT	98.03±0.99	90.00±6.60	97.46±1.78	96.03±0.04	89.87±10.74	87.51±0.70
G232E	69.36±13.95	23.87±5.84	82.28±7.16	87.33±2.83	68.75±8.39	65.13±10.23
Q294P	61.42±5.54	38.04±12.32	90.46±2.62	83.31±0.58	86.23±15.89	61.33±5.66
R386K	38.24±5.75	38.74±5.83	87.26±0.11	72.81±4.51	85.31±10.04	61.19±9.56
R482L	91.44±4.86	94.72±3.70	96.79±0.96	95.35±4.74	86.54±8.16	95.37±0.89

Values represent percentages of cells (mean ± SD) showing normal levels of HP1α or HP1β proteins, calculated from three independent experiments in which n = 75 transfected cells were analyzed in each experiment.

**Figure 2:**
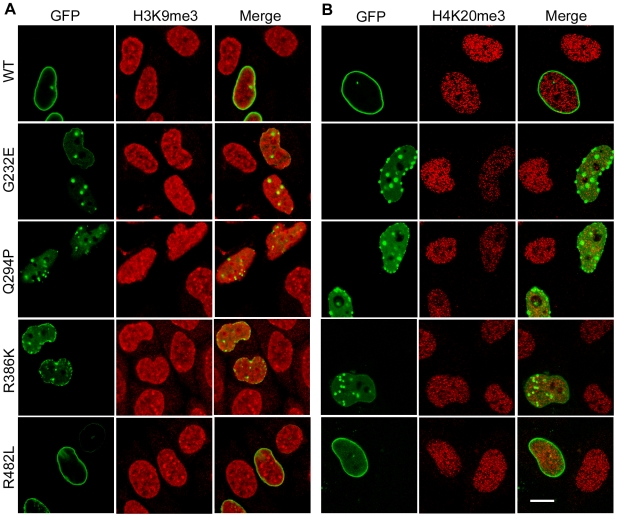
Localization of methylated histones in cells expressing lamin mutants. (**A,B**) HeLa cells were transiently transfected with the indicated GFP-tagged lamin constructs and stained with antibodies to H3K9me3 or H4K20me3 respectively. Bar, 10 µm.

As HP1α and β proteins bind to histone H3 trimethylated at lysine 9 (H3K9me3), which is a molecular mark of constitutive heterochromatin and is associated with silenced genes [27], we examined the localization of H3K9me3 in cells expressing GFP-lamin A constructs (Figure 2A). There were no major changes in the distribution or levels of H3K9me3 in G232E, Q294P or R386K-expressing cells, though small changes in distribution could not be ruled out. Histone H4 trimethylated at lysine 20 (H4K20me3), another marker for pericentric heterochromatin [28], showed slightly reduced staining in cells expressing these mutants (Figure 2B), but some variability in H4K20me3 staining was also observed in untransfected cells.

**Figure 3:**
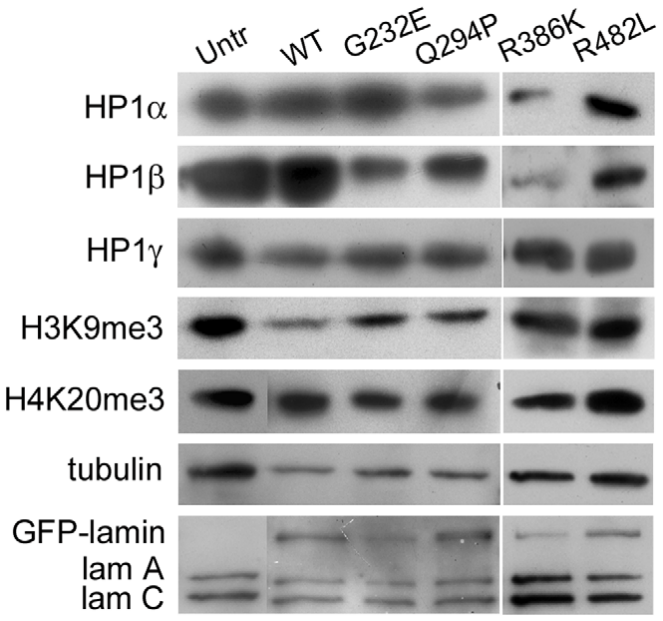
Expression of chromatin markers in HeLa cells expressing lamin mutants. Lysates of sorted, transfected cells and untransfected cells (∼50,000 cells per sample) were analyzed by western blotting with antibodies to the indicated proteins. The results shown are representative of three independent experiments.

The above results were substantiated by western blot analysis of cell lysates from FACS-sorted cells (Figure 3) followed by semi-quantitative analysis by scanning the blots and calculating the intensities normalized to tubulin. In comparison with untransfected cells, HP1β levels were decreased substantially in cells expressing G232E (22%), Q294P (44%) or R386K (26%) but not in cells expressing wild-type GFP-lamin A or R482L (∼90%). HP1α was significantly reduced in cells expressing R386K (48%) whereas HP1γ was not affected by any of the mutants (90–100%). The levels of H3K9me3 and H4K20me3 were also not significantly altered. Analysis of samples with anti-lamin A/C antibody indicated that the GFP constructs were expressed at levels comparable to endogenous lamin A under our experimental conditions of 24 h of transfection. Under these conditions, abnormal lamina assembly due to overexpression of the wild-type lamin A construct was observed in less than 10% of cells, as reported earlier [22]. The above immunofluorescence and western blot data thus indicate that the expression of lamin mutants G232E, Q294P or R386K results in the depletion of HP1 isoforms, but this does not cause major alterations in the organization of heterochromatic foci.

### Lamin A Mutants Induce Proteasomal Degradation of HP1α and β

**Figure 4:**
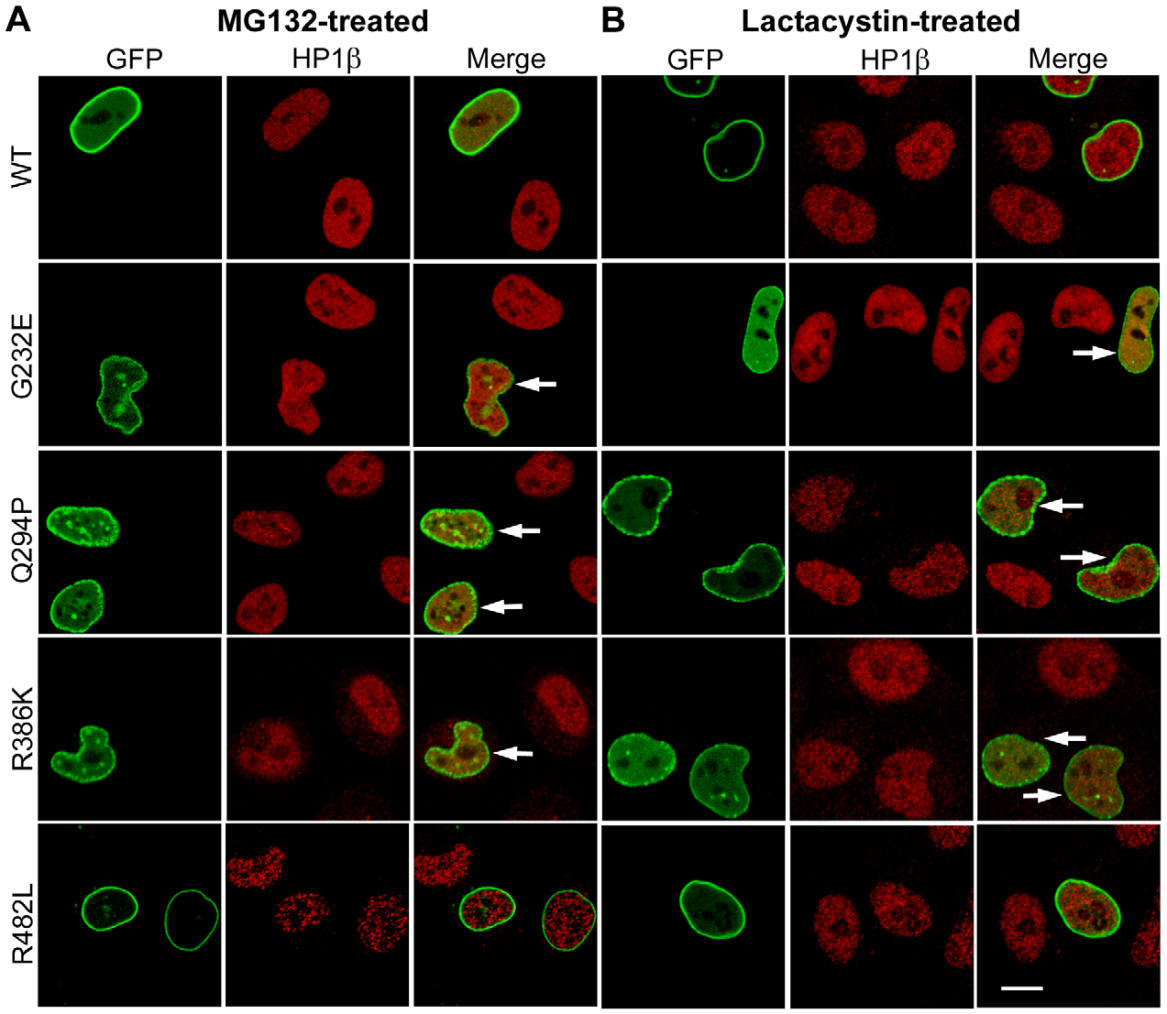
Restoration of HP1β after treatment with proteasomal inhibitors. HeLa cells transiently transfected with lamin constructs were treated with 6 µM MG132 or 10 µM lactacystin and stained with antibodies to HP1β. Arrows indicate transfected cells showing restoration of HP1β. Bar, 10 µM.

We next examined the possibility that depletion of HP1α and β might be due to proteasomal degradation by checking the effects of proteasomal inhibitors. We observed that addition of MG132 led to restoration of HP1α and β levels in transfected cells. As shown in Table 1, the percentage of G232E, Q294P or R386K-expressing cells that were positive for HP1α or β was substantially higher after MG132 treatment. Treatment with another proteasomal inhibitor, lactacystin also restored HP1 expression in the majority of cells expressing these mutants (Table 1). These data suggest that lamin A mutants induce proteasomal degradation of HP1α and HP1β. Interestingly, G232E and, to a lesser extent, Q294P and R386K were localized at the nuclear periphery after treatment with proteasomal inhibitors (Table 2). Large nuclear aggregates were reduced in number though the peripheral localization of these mutants was discontinuous and comprised of small aggregates (Figure 4). This was accompanied by partial improvement in the nuclear morphology of these cells. It was subsequently confirmed by real-time RT-PCR analysis that the transcript levels of HP1 isoforms were not altered by expression of lamin mutants (see Table 3).

**Table 2:** Effect of proteasomal inhibitors on localization of GFP-lamin A constructs.

Construct	Untreated	MG132-treated	Lactacystin-treated
WT	96.35±1.71	98.07±1.65	93.89±6.62
G232E	19.62±6.09	83.22±6.11	73.20±9.07
Q294P	9.09±2.21	69.77±5.16	46.20±6.40
R386K	15.41±5.11	61.01±6.40	39.76±8.57
R482L	95.39±2.77	97.41±1.81	92.61±7.18

Values represent percentages of cells (mean ± SD) showing localization of GFP-lamin A constructs at the nuclear rim, calculated from four independent experiments in which n = 75 transfected cells were analyzed in each experiment.

**Table 3:** Real-time PCR assays with stably transfected cells.

Gene	Function	GFP		Wild-type	G232E	R386K
CAND1	Cul1 binding protein	1.74±0.42		1.71±0.34	1.05±0.21	1.23±0.24
Cullin1	RING ligase core	1.20±0.32		1.32±0.34	−1.28±0.21	1.18±0.19
Cullin2	RING ligase core	1.20±0.10		1.46±0.33	1.47±0.30	1.51±0.31
Cullin4A	RING ligase core	1.37±0.21		1.59±0.25	−1.09±0.23	1.08±0.21
Cullin4B	RING ligase core	2.21±0.47		1.90±0.47	2.16±0.31	1.51±0.63
Cullin7	RING ligase core	−1.21±0.21		−1.22±0.10	−1.32±0.28	1.41±0.26
DZIP 3	DNA damage	1.12±0.13		−1.36±0.26	1.30±0.16	1.32±0.22
FBX04	Substrate binding	−1.60±0.30		−1.56±0.52	1.34±0.33	−1.22±0.16
FBXW10	Substrate binding	1.71±0.43		1.66±0.70	5.64±0.49	2.35±0.44
HECW2	HECT ligase	−1.47±0.31		1.55±0.35	2.93±0.37	1.86±0.60
RBX1	RING sub-unit	−1.24±0.22		1.10±0.38	−1.17±0.35	1.25±0.34
RNF123	RING finger protein	−1.33±0.12		−1.59±0.58	−1.59±0.49	3.77±0.69
RNF148	RING finger protein	1.57±0.31		1.29±0.31	1.47±0.29	1.43±0.38
SKP1	Adaptor	1.14±0.33		1.50±0.24	−1.07±0.23	1.47±0.17
UBE2G2	E2 enzyme	−1.64±0.27		−1.88±0.56	−1.84±0.55	−1.39±0.24
UBE2J1	E2 enzyme	−1.21±0.13		−1.15±0.18	1.32±0.38	1.17±0.19
UBE2L3	E2 enzyme	1.50±0.59		1.46±0.34	1.54±0.25	1.54±0.29
UBE2M	E2 enzyme	−1.24±0.37		1.40±0.36	−1.38±0.17	−1.29±0.26
UBE2S	E2 enzyme	2.62±0.45	1.63±0.49		1.37±0.34	1.86±0.57
UBE4B	Ubiquitination factor	1.51±0.31	1.44±0.29		1.37±0.32	1.57±0.29
HP1α	Heterochromatin	1.57±0.49	1.43±0.37		1.58±0.30	1.81±0.45
HP1β	Heterochromatin	−1.50±0.34	−1.75±0.56		−1.54±0.34	−1.67±0.39
HP1γ	Heterochromatin	1.57±0.44	1.28±0.23		1.46±0.39	1.42±0.35

Values indicate fold-change (up or down regulation) expressed as mean ± SD calculated from three independent experiments and normalized to untransfected HeLa cells.

### Lamin A Mutants Assemble Stably at the Nuclear Periphery after MG132 Treatment

**Figure 5:**
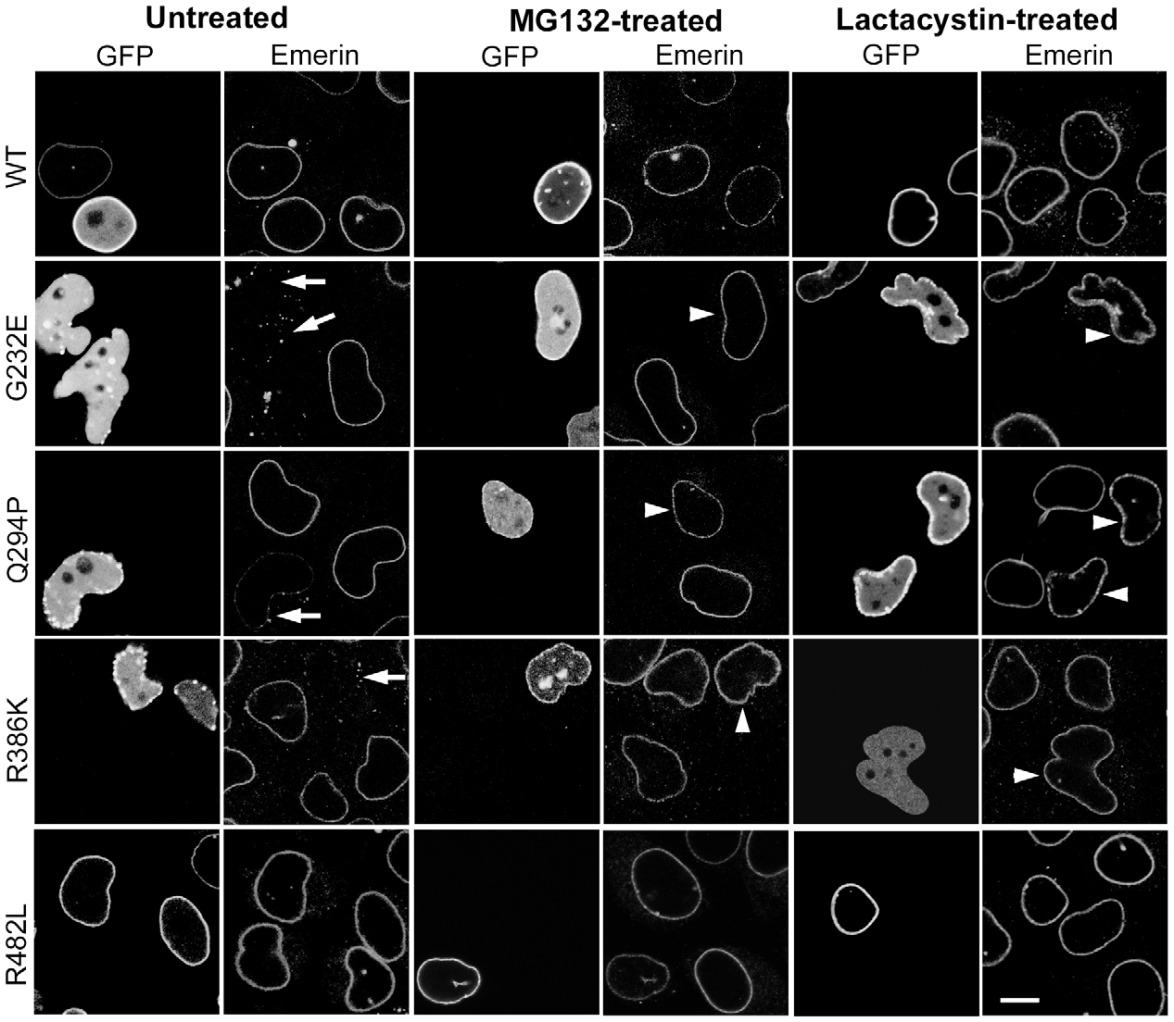
Restoration of emerin after treatment with proteasomal inhibitors. HeLa cells transfected with lamin constructs were treated with MG132 or lactacystin and stained with an antibody to emerin. Arrows indicate dispersed emerin staining; arrowheads point to restored emerin localization. Bar, 10 µM.

As we had noted that lamin mutants were localized at the nuclear periphery after treatment with proteasomal inhibitors, we sought to determine the stability of this structure. Staining of cells with an antibody to emerin, a lamin-binding protein localized in the inner nuclear membrane, indicated a depletion of emerin at the rim and a dispersed, cytoplasmic staining in untreated cells expressing G232E, Q294P or R386K, as observed in earlier studies with these constructs [22] as well as with other lamin A mutants [16], [19], [20]. Emerin was correctly localized to the nuclear rim in cells treated with MG132 or lactacystin (Figure 5), suggesting improvement in stable associations between the lamina and emerin. From this data we infer that inhibition of proteasomal activity allows lamin mutants to assemble into a more stable structure at the nuclear rim.

**Figure 6:**
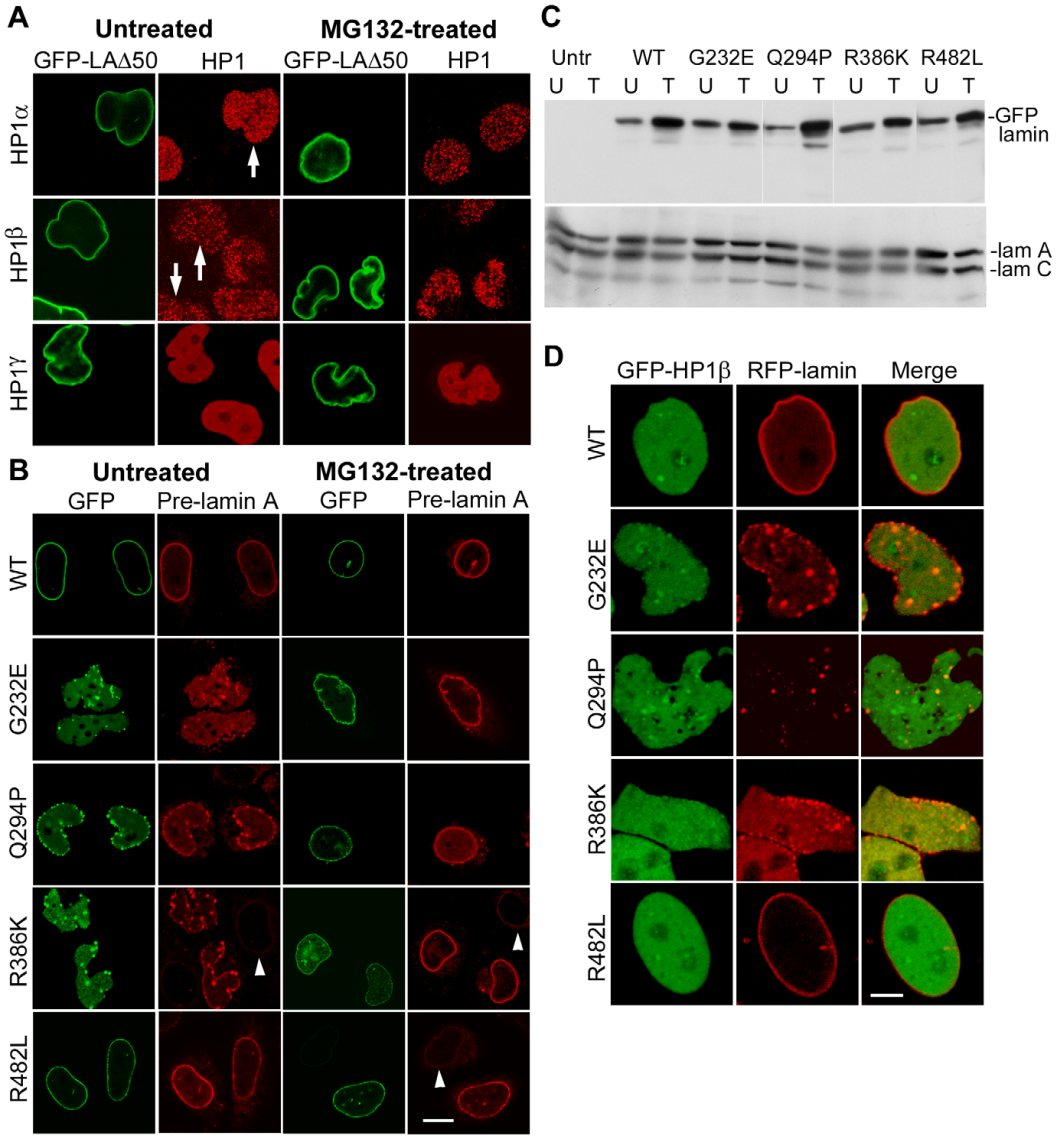
Effects of MG132 on lamin A processing. (**A**) Localization of LAΔ50. HeLa cells transfected with GFP-tagged LAΔ50 were treated with MG132 and stained with antibodies to HP1α, β or γ. Arrows indicate untreated transfected cells showing retention of HP1α or β. (**B**) Localization of pre-lamin A. HeLa cells transfected with lamin constructs were treated with MG132 and stained with an antibody to pre-lamin A. Arrowheads point to weak staining of untransfected cells. (**C**) Lamin profile after treatment with MG132. HeLa cells transfected with lamin constructs and untransfected cells were treated with MG132 and analyzed by western blotting with antibodies to GFP (upper blot) and lamin A/C (lower blot); U, untreated and T, treated with MG132. (**D**) Localization of GFP-HP1β. HeLa cells were co-transfected with GFP-HP1β and RFP-tagged lamin constructs. Bar, 10 µm in panels A and B, 5 µm in panel D.

To investigate whether factors other than restoration of emerin might be involved in location of lamin mutants at the nuclear periphery, the following experiments were carried out. We firstly analyzed the effects of MG132 on cells expressing LAΔ50, as this relocation could be due to MG132-induced changes in C-terminal farnesylation or processing. A number of studies have shown that the lobulated nuclear morphology of cells expressing LAΔ50 can be improved by drugs that block farnesylation of the C-terminus [12], [13], [29]–[32]. However, the aberrant nuclear morphology of LAΔ50-expressing cells was not altered, suggesting that C-terminal farnesylation was not affected by MG132 (Figure 6A). Interestingly, there were no significant changes in the levels of HP1α or β upon LAΔ50 expression. Secondly, we determined whether MG132 treatment altered the levels of pre-lamin A in cells expressing lamin A mutants, by staining with an antibody specific to the unprocessed C-terminus of pre-lamin A. There were no significant differences in the levels of pre-lamin A staining in treated and untreated cells expressing G232E, Q294P or R386K, though these mutants were located at the periphery after treatment with MG132 as expected (Figure 6B). Transfected cells expressed higher amounts of unprocessed wild-type and mutant GFP-lamins compared to endogenous pre-lamin A, which was probably due to saturation of the processing machinery by these exogenously expressed full-length lamin constructs, consistent with an earlier report [33]. We confirmed by western blot analysis that the electrophoretic mobilities of endogenous lamin A/C or GFP-lamin A in transfected cells were not altered after MG132 treatment of cells; although levels of endogenous lamin A/C were not affected, there was an increase in the amounts of all GFP-lamin constructs after MG132 treatment, which could be attributed to their reduced proteolysis (Figure 6C). The above results suggest that inhibition of the proteasomal system by MG132 does not affect farnesylation or processing of the C-terminus of lamin A mutant constructs.

We also assessed the effects of ectopically expressed HP1β on the nuclear morphology of G232E, Q294P or R386K-expressing cells. When GFP-HP1β was co-transfected with mRFP-lamin A constructs into HeLa cells, mutant lamin A aggregates persisted in the nuclear interior, nuclear morphology remained distorted and G232E, Q294P or R386K were not localized to the nuclear periphery (Figure 6D). Occasional GFP-HP1β foci were colocalized with mutant lamin aggregates. These data demonstrate that restoration of HP1β is not sufficient to relocate mutant lamins to the nuclear rim.

### HP1α and β are Less Stable than HP1γ

**Figure 7:**
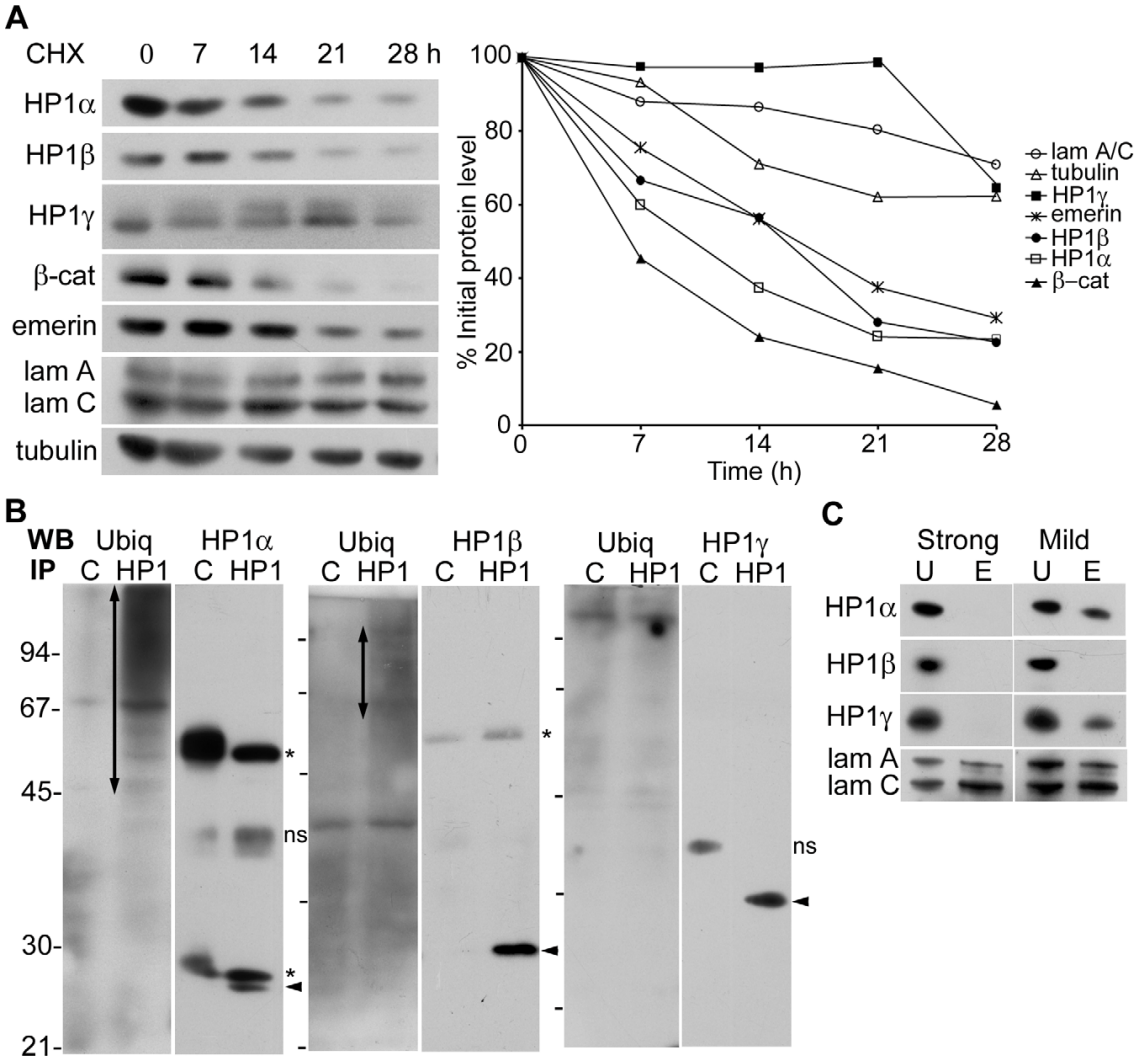
Stability and solubility of HP1 isoforms. (**A**) Protein turnover in the presence of cycloheximide. HeLa cells were treated with cycloheximide (CHX) for 0-28 h and levels of indicated proteins were determined by western blot analysis followed by a semi-quantitative analysis by scanning of blots. Graphical data is representative of two separate experiments. (**B**) Basal levels of ubiquitination of HP1 isoforms. Individual HP1 isoforms were immunoprecipitated from HeLa cell lysates and analyzed by western blotting with antibodies to ubiquitin as well as to HP1α, β or γ. Vertical arrows point to multi-ubiquitinated forms of HP1α or β; arrowheads, HP1; ns, non-specific bands; asterisk, IgG light/heavy chain; molecular mass markers: phosphorylase b, 94 kD; albumin, 67 kD; ovalbumin, 45 kD; carbonic anhydrase, 30 kD; trypsin inhibitor, 21 kD. (**C**) Matrix association of HP1 isoforms. HeLa cell monolayers were extracted with Triton X-100, Tween-20 and sodium deoxycholate (strong) or with Triton X-100 only (mild) followed by nuclease digestion and salt extraction. The extracted pellet, E and unextracted cell lysates, U were analyzed by western blotting with antibodies to HP1 isoforms and lamin A/C.

The specific degradation of HP1α and β but not HP1γ by lamin misexpression prompted us to compare the stability and solubility properties of the individual isoforms. An initial assessment of their inherent stability was obtained by analysis of basal turnover rates using the protein synthesis inhibitor cycloheximide. HeLa cells were treated with cycloheximide in a time-course experiment and levels of HP1 isoforms were analyzed by western blotting (Figure 7A). We observed that both HP1α and β were degraded more rapidly than HP1γ, which was highly stable. Lamins A/C and tubulin were also very stable during the course of the experiment while emerin was comparatively less stable. The positive control, β-catenin was rapidly degraded as expected. Next, we assessed the ubiquitination levels of the endogenous HP1 isoforms by immunoprecipitation of cell lysates with antibodies to HP1 proteins followed by western blot analysis of the immunoprecipitates with antibody to ubiquitin. As shown in Figure 7B, both HP1α and β immunoprecipitates showed higher levels of ubiquitination compared to HP1γ (which was similar to the control immunoprecipitate). We then checked the binding of the individual HP1 isoforms to nucleoskeletal fractions of HeLa cells prepared by two different procedures. All three isoforms were completely solubilized by extraction of nuclei under strong conditions with a combination of detergents to deplete most soluble proteins, but HP1α and HP1γ were bound to the nucleoskeleton under milder conditions (Figure 7C). Lamins A and C were retained in the nucleoskeletal fraction in both procedures. The above findings indicate that, in addition to well-documented differences in association with heterochromatin and euchromatin, HP1α and β have a faster turnover rate and higher levels of basal ubiquitination than HP1γ. Furthermore, HP1β is not as strongly associated with the nucleoskeleton as HP1α and HP1γ.

### Lamin Mutants Activate a Specific F-Box Protein

The induction of proteasomal degradation by lamin mutants prompted us to check whether specific components of the ubiquitin-proteasome system were activated by the mutants. This system is comprised of an E1 ubiquitin activating enzyme, an E2 ubiquitin conjugating enzyme and an E3 ubiquitin ligase. Substrate specificity is conferred by the large variety of E3 enzymes that can recognize distinct substrates through specific domains or modules [34]. In order to identify differentially regulated transcripts in this pathway, RNA was analyzed from stably transfected HeLa cells harbouring wild-type GFP-lamin A and representative mutants G232E and R386K. Cells transfected with the GFP vector alone and untransfected cells were used as controls. After an initial analysis of transcripts of 85 genes by a PCR array of different components of the ubiquitination pathway (SABioscience, data not shown), we chose 20 genes that had shown at least 1.5-fold changes in levels for detailed study. Transcripts were quantitated by real-time RT-PCR analysis and the results are shown in Table 3. A substantial upregulation of 5.6-fold in the presence of G232E and 2.3-fold in the presence of R386K mutant was observed with a gene coding for an F-box family protein, termed FBXW10. As F-box proteins are components of the RING family of E3s [35], other members of these complexes such as the cullins, CAND1, RBX1 and SKP1 were also analyzed, but transcript levels of these genes were not significantly affected by lamin mutants. We noted that levels of cullin 4, which has been implicated in heterochromatin formation [36], were not altered. On the other hand, moderate induction of the HECW2 ligase and RNF123 RING finger protein was observed with G232E and R386K-expressing cells respectively (but not both).

**Figure 8:**
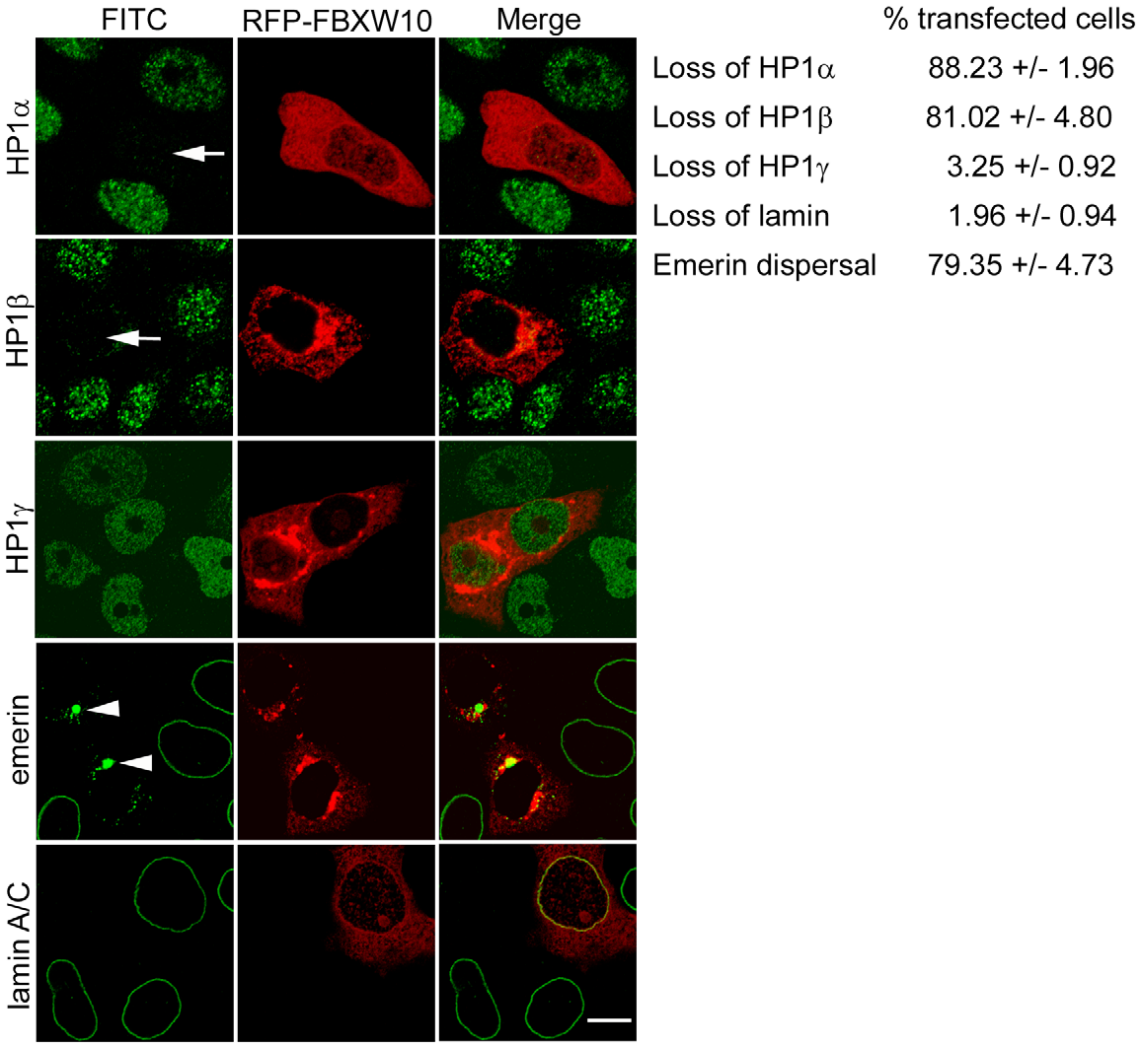
Effects of FBXW10 on HP1 isoforms and nuclear envelope proteins. HeLa cells transfected with RFP-tagged FBXW10 were stained with antibodies to HP1 isoforms, emerin or lamin A. Arrows indicate transfected cells depleted of HP1α or β; arrowheads point to dispersed emerin localization. Values represent mean ± SD, calculated from three independent experiments in which n = 75 transfected cells were analyzed in each experiment. Bar, 10 µM.

As the F-box proteins are important determinants of substrate specificity in the ubiquitination process, we carried out further experiments with FBXW10. The gene was cloned by PCR and expressed as an epitope-tagged protein in HeLa cells. RFP-FBXW10 was located mostly in the cytoplasm of transfected cells. Interestingly, expression of FBXW10 resulted in the depletion of HP1α and β in 80–90% of cells without alteration of HP1γ levels (Figure 8). In the majority of cells (∼80%), emerin was dispersed in the cytoplasm while the remainder showed peripheral localization of emerin. However, nuclear rim staining of endogenous lamin A was not altered, suggesting that expression of FBXW10 and depletion of HP1 do not directly cause lamin mislocalization. We conclude from these experiments that expression of a specific F-box protein that is induced by lamin mutants results in depletion of HP1α and β and disruption of emerin localization.

## Discussion

In this study, we report that HP1α and β undergo proteasomal degradation in cells expressing lamin mutants that predominantly form intranuclear aggregates. Treatment with proteasomal inhibitors restores HP1α and HP1β, and locates G232E, Q294P and R386K together with emerin to the nuclear periphery. Analysis of components of the ubiquitination pathway indicates that expression of the F-box protein FBXW10 is highly induced in mutant-expressing cells, and ectopic expression of FBXW10 results in specific depletion of HP1α and β. The implications of our results are discussed with respect to the effects of lamin misexpression on chromatin organization, lamina assembly and protein ubiquitination.

### Lamins and Chromatin Organization

At the nuclear periphery, the lamina is closely associated with inner nuclear membrane proteins such as lamin B receptor and emerin, and the chromatin-binding protein BAF [7], [8]. Lamin B receptor interacts with B-type lamins and HP1 while emerin binds to both A-type lamins and BAF. In the nuclear interior, LAP2α associates with chromatin and intranuclear lamin A [37], [38]. Thus lamins associate with chromatin either directly or indirectly through bridging proteins. The importance of the lamina network for proper chromatin organization is supported by the observation of heterochromatin fragmentation in *Lmna^−/−^* mouse cardiomyocytes [39], and loss of heterochromatin and occurrence of developmental defects in lamin null mutants in *C. elegans* [40]. Furthermore, genome-wide mapping studies have revealed interactions of B-type lamins with the genome [41], [42], and a number of studies suggest that transcriptionally silent chromatin is preferentially located at the nuclear periphery [43].

Expression of LAΔ50 or higher levels of pre-lamin A is toxic to cells due to persistence of farnesylated lamins, and results in changes in heterochromatin organization accompanied by alterations in histone methylation and HP1 distribution [11]–[15], [29], [32], [44], [45]. Our studies have shown that expression of lamin A rod domain mutants, but not LAΔ50, results in proteolytic degradation of HP1α and β without causing major changes in the levels or distribution of methylated histones associated with pericentric heterochromatin. The targeting of HP1 isoforms for proteasomal degradation in these cells thus provides an additional mechanism through which lamin mutants can alter chromatin organization that is distinct from the toxicity of LAΔ50 or overexpressed pre-lamin A. A recent study has identified another progeria mutation, E145K that is highly disruptive of nuclear structure but does not respond to treatment with a farnesyl transferase inhibitor [46]. It would be interesting to know whether the extensive defects in chromatin observed in this system are due to proteasomal degradation of chromatin proteins.

### Stable Assembly of Lamina at Nuclear Periphery

Wild-type lamins form highly stable polymeric species at the nuclear periphery while several rod domain lamin A mutants form less stable aggregates in the nucleoplasm [47]–[50]. We have observed that lamin A mutants are localized to the nuclear periphery after treatment with proteasomal inhibitors. The inhibition of proteasomal activity might induce stable association of lamin mutants at the nuclear periphery through direct effects on lamin A structure and assembly and/or indirect effects on the stability of peripheral lamin-binding proteins, as the rim localization of lamin A is highly sensitive to C-terminal post-translational modifications as well as to localization of inner membrane-associated proteins such as emerin [51], [52]. Since the peripheral localization of G232E, Q294P and R386K in the presence of proteasomal inhibitors was accompanied by restoration of emerin at the nuclear periphery, our data emphasize the importance of stable interactions between lamins and inner membrane proteins for appropriate nuclear rim localization of lamins. It is not clear at present whether additional factors might be involved in the stable assembly of lamin mutants at the nuclear periphery.

### HP1 and Chromatin Organization

Mammalian HP1 isoforms are specifically enriched in heterochromatin or euchromatin and their domains of localization in turn influence their activities [25]. The localization and mode of action of HP1 depend on the sequence of the DNA, association with interacting proteins and the cell type involved, in addition to binding interactions with H3K9me3 [26], [53]. Our data with lamin A/C mutants imply that a normal lamina is also necessary for stable expression of HP1α and HP1β, as aberrant lamina assembly reduces levels of these isoforms by induction of their proteasomal degradation. Although we have not observed significant alterations in methylated histones or changes in pericentric chromatin domains in HeLa cells expressing lamin mutants, which is consistent with previous reports that depletion of HP1α or β isoforms does not affect maintenance of pericentric chromatin or levels of H3K9me3 associated with such domains [54], [55], the possibility exists that loss of HP1 may alter chromatin function and regulation of specific genes, especially in view of the perinatal lethality of HP1β knock-out mice [56] and a role for HP1β in initiating a DNA damage response [57].

### Protein Ubiquitination and Lamina Organization

The cullin-based E3 ubiquitin ligases, also known as RING E3 ubiquitin ligases, perform most of the targeted protein ubiquitination that occurs in eukaryotic cells [34]. These are modular ligases and are comprised of a cullin-based core and a substrate specificity module. The cullin proteins (Cul 1–7) have diverse functions such as cell cycle regulation, DNA repair and regulation of developmental processes. In the Cul 1 complex, which is the canonical RING ligase, a single SKP1 adaptor protein is bound to a member of the F-box family of substrate binding proteins, of which there are 68 members in the human genome [34], [35]. However, target substrates have been identified for only a few F-box proteins. We have observed that a specific F-box protein, FBXW10 is upregulated on expression of lamin mutants that form abnormal nuclear aggregates. Overexpression of FBXW10 resulted in substantial depletion of HP1α and β, but not HP1γ. Endogenous lamin A levels were not altered in the presence of ectopic FBXW10, but the majority of cells showed cytoplasmic dispersal of emerin, suggesting that nuclear envelope structure is susceptible to the increased degradation triggered by FBXW10.

A role for misexpression of lamin A in activating the ubiquitin-proteasome system is supported by the finding that in fibroblasts from a human patient with a homozygous *LMNA* nonsense mutation (Y259X) which leads to absence of lamin A, the integral membrane proteins emerin and nesprin-1α are mislocalized to the ER and subsequently degraded by the proteasomal machinery [58]. Also, proteasomal degradation of retinoblastoma protein is increased in mouse cells derived from lamin A knock-out mice [59]. However, these studies have not addressed the mechanism of proteasomal degradation by lamin misexpression.

In summary, our study shows for the first time that inappropriate assembly of lamin A/C leads to proteasomal degradation of specific heterochromatin proteins by activation of a distinct F-box protein. Thus ubiquitin-mediated proteasomal degradation of essential nuclear proteins may afford a distinct mechanism for the deleterious effects of disease-causing lamin mutants. Future studies should give insights into the precise mechanism of action of FBXW10, as well as the involvement of other components of the E3 ubiquitin ligase systems in these processes.

## Materials and Methods

### Plasmid Constructs

Wild-type GFP-lamin A and lamin mutant constructs have been described earlier [22], [60]. Lamin inserts from these pEGFP plasmids were recloned into an mRFP mammalian expression vector (Clontech) for specific experiments. A GFP-HPIβ expression vector was kindly provided by Peter Hemmerich (Institute of Molecular Technology, Jena, Germany). For cloning of full length human FBXW10 (accession no. NM_031456.3), total cDNA was obtained from HeLa RNA by reverse transcription using Superscript II reverse transcriptase kit (Invitrogen) as per the manufacturer's instructions. The 3-kb FBXW10 gene was initially amplified by PCR as three separate fragments, using Phusion™ high fidelity DNA polymerase (Finnzymes) as per the manufacturer's instructions and with the appropriate primers listed below, followed by cloning into pMOS vector. Then the middle and 3′ fragments were reamplified by overlap PCR to give a single fragment which was ligated to the 5′ fragment after appropriate enzyme digestion and cloned into XhoI, BamHI-digested pEGFP-C1 mammalian expression vector (Clontech) and recloned into an mRFP mammalian expression vector (Clontech). The gene construct was verified by automated DNA sequence analysis. The PCR primers used were as follows (added restriction sites in small case):

(i) 5′ fragment, forward: 5′ccgctcgagcgATGGAAAACCTGGAATCAAGGCTC3′; reverse: 5′CTCTTGGGCTGTGAACCTGAAT3′; (ii) middle fragment, forward: 5′CCTGTGGACTGCATACCAGAACGA3′; reverse: 5′GCTAGCATGCGGTTTGGTTT3′; (iii) 3′ fragment, forward: 5′CCCCAGCCCATGATTATCC3′; reverse: 5′cgggatcccgTCCCAAGGCTGGTTTAGAT3′.

### Cell Culture, DNA Transfection, Drug Treatment and Nuclear Extractions

HeLa cells were routinely grown in DMEM supplemented with 10% FBS at 37°C in a humidified atmosphere containing 5% CO_2_. Plasmid constructs were transiently transfected into HeLa cells for 24 h using Lipofectamine 2000 (Invitrogen) according to the manufacturer's instructions. Stably transfected cells expressing wild-type, G232E or R386K GFP-lamin constructs were obtained by blasticidin selection of HeLa cells transfected with pEGFP lamin plasmids in which the neomycin resistance cassette was replaced by a blasticidin resistance cassette. A similar strategy was used for isolating cells stably transfected with pEGFP control vector. Alternately, cells transiently expressing wild-type or mutant GFP-lamin A were enriched by sorting for GFP expression on a Moflo cell sorter at 488 nm excitation wavelength (which enriched transfected cells to ∼99%) for western blot analysis, as the above strategy was not successful for obtaining cells stably expressing Q294P. For treatment with proteasomal inhibitors, cells were incubated with 6 µM MG132 (Calbiochem) or 10 µM lactacystin (Calbiochem) for 18 h, starting at 6 h after transient transfection of plasmids. For protein half-life studies, cells were treated with 100 µg/ml cycloheximide for 0–28 h. Nuclear extractions of cell monolayers were carried out by a mild extraction protocol using Triton X-100 as described by De Conto *et al.* [61] or by a stronger extraction procedure requiring Triton X-100, deoxycholate and Tween-20 as described by Nickerson *et al.* [62].

### Antibodies, Western Blot Analysis and Immunoprecipitations

Polyclonal antibodies to lamin A/C or the C-terminus of pre-lamin A and a mouse mAb to tubulin were obtained from Santa Cruz Biotechnology. Polyclonal antibodies or mouse mAbs to HP1α, β and γ were obtained from Upstate or Chemicon. Polyclonal antibodies to H4K20me3 and H3K9me3 were from Abcam and Upstate respectively. Mouse mAbs to emerin (clone 4G5) and ubiquitin were from Novocastra Laboratories and Calbiochem respectively. For western blot analysis, samples (∼50,000 cells per sample) were lysed in Laemmli's sample buffer, boiled and electrophoresed through SDS-10% polyacrylamide gels. Gels were electroblotted onto PVDF membrane filters and blocked overnight in 5% BLOTTO in Tris-buffered saline containing 0.1% Tween-20. Filters were incubated with primary antibody for 2 h, followed by peroxidase conjugated-secondary antibody in Tris-buffered saline containing 0.1% Tween-20 for 1 h. Bound antibody was visualized using a chemiluminescence kit from Roche Applied Science. For immunoprecipitation assays, cells were lysed in cold RIPA buffer containing 20 mM Tris.HCl, pH 7.4, 150 mM NaCl, 1% NP-40, 1 mM PMSF, 20 mM NaF, 1 mM sodium vanadate and protease inhibitors. The precleared samples were immunoprecipitated with primary antibody followed by protein A-Sepharose beads and the bound proteins were analyzed by western blotting.

### Immunofluorescence Microscopy

Transfected HeLa cells were washed with PBS and then fixed with 4% formaldehyde in phosphate-buffered saline (PBS) for 10 min followed by treatment with 0.5% (vol/vol) Triton X-100 for 6 min at room temperature. Cells were then incubated with 0.5% gelatin in PBS for 1 h followed by incubation with primary antibody for 1 h and then Cy3- or FITC-conjugated secondary antibody for 1 h at room temperature. Samples were mounted in Vectashield (Vector Laboratories) containing 1 µg/ml DAPI. Fluorescence microscopy of fixed cells was performed on an LSM510 META or LSM510 META/NLO confocal microscope. DAPI staining was routinely viewed in the transmission mode. Images were analyzed with LSM 510 META software and assembled using Adobe Photoshop.

### Quantitative RT-PCR Analysis

Total RNA was extracted from stably transfected HeLa cells and 1 µg of RNA was reverse transcribed using Superscript II reverse transcriptase kit (Invitrogen) as per the manufacturer's instructions. Amplification of PCR products was quantitated using SyBR green dye (ABI), and fluorescence was monitored on an ABI prism 7900HT sequence detection system. Melting curve analysis was done for each amplicon. The 2^−ΔΔCt^ method was used for quantitation with HPRT1 (hypoxanthine phosphoribosyl transferase 1) as endogenous control. The analysis for each gene was done in triplicate and three independent biological replicates were performed. Gene expression in transfected cells was expressed as fold change in comparison with untransfected HeLa cells. The gene specific primers used for the analysis are given in Table S1.

## Supporting Information

Table S1 List of primers used for real-time PCR analysis.
(0.05 MB DOC)
