# Supplementary material for: Lamin A Rod Domain Mutants Target Heterochromatin Protein 1α and β for Proteasomal Degradation by Activation of F-Box Protein, FBXW10
Source: PLoS One. 2010 May 13;5(5):e10620. doi: 10.1371/journal.pone.0010620 (PMC2869352; doi:10.1371/journal.pone.0010620)
Supplement: Table S1 — List of primers used for real-time PCR analysis. (0.05 MB DOC) [file pone.0010620.s001.doc]

Table 1S List of primers used for real time PCR analysis

| No. | Gene | Forward primer 5’ to 3’ | Reverse primer 5’ to 3’ | Amplicon (bp) |
| --- | --- | --- | --- | --- |
| 1 | CAND1 | TGGGTTCTGACTTGCCTAATACAC | CCAGAACAGGCCTCAAATCTATCT | 135 |
| 2 | Cul1 | GCCCTGGTAATGTCTGCATTC | ACAGTATCGAGCCAGCAACTC | 144 |
| 3 | Cul2 | GCGGACCTTCAGTATGGCTATG | ATTTCTCGGAGCAGCATTCG | 140 |
| 4 | Cul4A | TCTTGGACCGCACCTATGTG | CTCGATCAGCAGTAGGATTCCAT | 140 |
| 5 | Cul4B | CATGAATGCGGAGCTGCTT | GCCCATTGTCAGGATATTCACA | 153 |
| 6 | Cul7 | GAAACCTGACGCGCTGTTG | CCCGAGGCCCAAACAGTT | 150 |
| 7 | DZIP3 | ATGGGAAGGAGCCAGTAATCC | TCCCCTGTTGCATCAACCA | 155 |
| 8 | FBXO4 | CGTCCTATGTATGGAGCTGTCACT | TGGGCAAAGTTCCTCTGAAGAC | 144 |
| 9 | FBXW10 | CCCCAGCCCATGATTATCC | GCTAGCATGCGGTTTGGTTT | 140 |
| 10 | HECW2 | ACACGGTGCTCCTCTCTTGAG | GCAGTGCACACGGATTCCT | 140 |
| 11 | HP1α | GAAAAACTTGGATTGCCCTGAG | GCGATATCATTGCTCTGCTCTCTC | 180 |
| 12 | HP1β | CCGTCGAGTGGTAAAGGGCAAAG | GCGCTTGCCTCCCTCTGATTTATC | 172 |
| 13 | HP1γ | GCTGGCAAAGAAAAAGATGGTAC | CATCAATTCTCCACTGCTGTCTGT | 168 |
| 14 | HPRT1 | GCCCTGGCGTCGTGATTA | CATCTCGAGCAAGACGTTCAGT | 144 |
| 15 | RBX1 | TAACCAGGCGTCCGCTACTT | CCACTCTCTGTTGTCCAATGGA | 130 |
| 16 | RNF123 | CTACCTGAACCGCATCTTTTCC | TCAACCTGTCCCTGGCTTTC | 147 |
| 17 | RNF148 | CTGGCCCTCATCGAACGT | TTCCGTCCCCTGGTGAGA | 141 |
| 18 | SKP1 | GTAGTGGCTTCGTCTTCGGTTT | TCTCTCCATCAGAACTCTGCAACT | 130 |
| 19 | UBE2G2 | GTGCAGAGTGTGGAGAAGATCCT | GGACGATCTGCTTGGCAATC | 142 |
| 20 | UBE2J1 | CTCCTAACGGCTAATGGTCGAT | ACCTATGGCTCCCTCTCCTTTT | 156 |
| 21 | UBE2L3 | CCAAGTAATCCAGTCCCTCATAGC | ACAGGTCGCTTTTCCCCATA | 153 |
| 22 | UBE2M | CAGAAGGACATAAACGAGCTGAAC | CTGAACACAAACTTCCCACTCTTG | 134 |
| 23 | UBE2S | CCCATATGCTGGAGGTCTGTTC | TGAGCACGTTGACGCAGATC | 140 |
| 24 | UBE4B | TGGAGGAGATAGTGGCCAAGA | AGGTGCCGCAGGATGATG | 154 |
